# Regulation of Stress-Activated Kinases in Response to Tacaribe Virus Infection and Its Implications for Viral Replication

**DOI:** 10.3390/v14092018

**Published:** 2022-09-12

**Authors:** Julia Holzerland, Lucie Fénéant, Allison Groseth

**Affiliations:** Laboratory for Arenavirus Biology, Institute of Molecular Virology and Cell Biology, Friedrich-Loeffler-Institut, 17493 Greifswald, Germany

**Keywords:** Tacaribe virus, apoptosis, kinase activation, kinase inhibitors, pathogenesis, p38, JNK

## Abstract

Arenaviruses include important zoonotic pathogens that cause hemorrhagic fever (e.g., Junín virus; JUNV) as well as other viruses that are closely related but apathogenic (e.g., Tacaribe virus; TCRV). We have found that, while TCRV and JUNV differ in their ability to induce apoptosis in infected cells, due to active inhibition of caspase activation by the JUNV nucleoprotein, both viruses trigger similar upstream pro-apoptotic signaling events, including the activation/phosphorylation of p53. In the case of TCRV, the pro-apoptotic factor Bad is also phosphorylated (leading to its inactivation). These events clearly implicate upstream kinases in regulating the induction of apoptosis. Consistent with this, here we show activation in TCRV-infected cells of the stress-activated protein kinases p38 and JNK, which are known to regulate p53 activation, as well as the downstream kinase MK2 and transcription factor c-Jun. We also observed the early transient activation of Akt, but not Erk. Importantly, the chemical inhibition of Akt, p38, JNK and c-Jun all dramatically reduced viral growth, even though we have shown that inhibition of apoptosis itself does not. This indicates that kinase activation is crucial for viral infection, independent of its downstream role in apoptosis regulation, a finding that has the potential to shed further light on the determinants of arenavirus pathogenesis, as well as to inform future therapeutic approaches.

## 1. Introduction

The arenavirus family includes many important viruses that can infect mammals, as well as a wide range of reptile-associated viruses that have been recently identified [1,2]. Those arenaviruses that infect mammals are maintained in rodent reservoir hosts, where they are persistent and do not cause any significant disease. In contrast, transmission to humans results in infections that can be quite severe and even result in hemorrhagic fever and/or neurological manifestations [3]. Of particular importance to public health is the highly pathogenic Old World (OW) arenavirus Lassa virus (LASV), which causes hemorrhagic fever (HF) in endemic regions of West Africa [4]. Similarly, highly pathogenic New World (NW) arenaviruses, for instance, the causative agent of Argentine HF, Junín virus (JUNV), pose a local disease threat in geographically restricted regions of South America that are defined by their rodent reservoir hosts [5]. However, among both the OW and NW arenaviruses there are a number of viruses that, despite being closely related to these highly virulent members, show little or no virulence in humans. Well-studied examples include Tacaribe virus (TCRV) and Pichinde virus (PICV) [6,7].

At present, little is known about the basis for these differences in pathogenicity between closely related arenaviruses, however, an increasing number of recent studies suggest the involvement of host cell responses, including the interferon response (reviewed in [8]), cytokine expression [9,10,11,12] and cell death regulation (reviewed in [13]). In particular, recent studies have demonstrated a connection between apoptosis induction and a non-pathogenic phenotype, revealing that both TCRV and the attenuated vaccine strain of JUNV (Candid#1) are able to induce late steps in the apoptotic signaling cascade (i.e., caspase activation and cell death), whereas virulent JUNV does not [14,15,16,17]. Interestingly, however, we have shown that this is due to the ability of JUNV to actively suppress caspase activation through the activity of its nucleoprotein (NP), which can serve as a decoy substrate for caspase cleavage [18]. Consistent with this model, our recent work characterizing the factors involved in the induction of intrinsic apoptosis during TCRV- and JUNV-infection showed striking similarities in the regulation of pro- and anti-apoptotic factors [17]. In particular, we observed phosphorylation of both p53 and Bad during TCRV-infection, which regulates their activation state and thus their ability to mediate the transcription of pro-apoptotic factors, or participate in the regulation of mitochondrial permeability, respectively [17]. However, while the presence of these specific phosphorylation events clearly suggests the involvement of kinase pathways in initiating apoptotic signaling [19,20,21,22], it is currently unknown which kinases/signaling pathways are responsible for these modifications. Furthermore, the fact that activation of these pathways appears to be common to both TCRV and JUNV infection, despite their different outcomes with respect to apoptotic cell death, suggests a more fundamental role of kinase activation in arenavirus biology.

Indeed, several previous studies have indicated the activation of different mitogen-activated protein kinases (MAPKs) in response to NW arenavirus infection, including some that are known to regulate p53 and Bad phosphorylation. For instance, the activation of the pro-survival kinase Erk has been shown during JUNV XJ13 (an attenuated strain belonging to the Candid#1 vaccine linage), TCRV and PICV infection [23,24,25]. In contrast, the Erk signaling pathway seems to be perturbed in response to LASV entry [26]. Interestingly, Erk signaling has been shown to be able to mediate both Bad and p53 phosphorylation in some contexts [27,28]. Another kinase that is also known to phosphorylate Bad is Akt [21], for which an early activation during JUNV XJ13 infection has also been demonstrated [29,30]. More recently, studies have also indicated that among the MAPKs, stress-activated protein kinases (SAPKs) may also play an important role. In particular, the activation of the SAPK p38 has been shown during PICV infection [31], as well as during JUNV XJ13 and Candid#1 infection [32,33]. Indeed, SAPKs such as p38 and JNK are known to be the major regulators involved in stress-response activated induction of apoptotic signaling via phosphorylation of p53 at various positions [28]. As such, they represent strong candidates to play a role in kinase-mediated p53 activation during TCRV-induced apoptosis.

To investigate a potential role for kinase activation in the induction of apoptosis (i.e., via Bad and p53 phosphorylation) during TCRV infection, we investigated the regulation of Erk, Akt, p38 and JNK, as well as the downstream kinase MK2 (a target of p38) and the AP-1 family transcription factor c-Jun (a target of JNK). Furthermore, we examined the relevance of these factors for viral infection using various inhibitors. Infection with TCRV revealed significant activation of MK2, p38, JNK and c-Jun late in infection, but also transient weak activation of Akt early after infection. Interestingly, we also saw that, unlike the inhibition of apoptosis itself [17], the inhibition of some of these factors strongly impairs virus growth. This demonstrates the crucial role of these kinases during arenavirus infection independent of their ability to induce apoptosis.

## 2. Materials and Methods

### 2.1. Cells

Vero76 (CCLV-RIE0228) and HEK293 (CCLV-RIE0197) cells were grown in Dulbecco’s Modified Eagle’s Medium (DMEM) supplemented with 10% fetal calf serum (FCS), 2 mM L-glutamine (Q), 100 U/mL penicillin and 100 μg/mL streptomycin (P/S; Thermo Fisher Scientific, Waltham, MA, USA) at 37 °C with 5% CO_2_.

### 2.2. Virus Infection and Plaque Assay

TCRV (strain TRVL-11573) was used to infect Vero76 and HEK293 cells with a confluence of 80–90% in 12-well plates. Infections were performed at a multiplicity of infection (MOI) of 0.1 and incubated for 60 min at 37 °C with 5% CO_2_. Subsequently, the inoculum was removed and replaced with DMEM containing 2% FCS and P/S. Supernatants were sampled at different time points 0 to 4 dpi, as indicated in the individual experiments. The resulting samples were processed for Western blot (as described below in Section 2.5), or analyzed for virus release by plaque assay.

Briefly, for plaque assay, virus supernatants were serially diluted in DMEM without FCS, and used to infect 100% confluent Vero76 cells in 12-well plates. Infections were allowed to proceed for 1 h at 37 °C after which the virus was removed and the wells were overlaid with 0.7% agarose in minimal essential medium (MEM) containing 2% FCS. Plates were incubated for 7 days and then stained with a crystal violet solution (10% formaldehyde; 0.1% crystal violet).

### 2.3. Chemical Treatments

In order to inhibit or activate kinases of interest during infection, Vero76 cells were incubated with the commercially available drugs (Tocris Bioscience, Bristol, UK; Sigma–Aldrich, St. Louis, MO, USA) listed in Table 1 at the indicated final concentrations in DMEM supplemented with 2% FCS and P/S. As controls, treatment with the same amount of DMSO used to prepare the drugs and infection without any treatment (Mock) were performed. For chemical treatment during the early phase of the infection, cells were pre-treated with the indicated drug for 2 h, followed by infection (performed as explained above in Section 2.2). After the infection, fresh medium containing the same drug was applied and supernatants were sampled from day 0 (i.e., directly after replacing the inoculum with fresh medium containing 2% FCS and P/S) up to day 3 post infection (pi), as indicated. To assess the impact of these drugs during the later phase of infection, TCRV infection was performed as described above and samples were taken on day 0 pi. After 2 days, supernatants were again sampled (day 2 pre), after which the chemicals were added to the infected wells at the indicated concentrations, but without replacing the medium. Four hours post addition of the chemical, samples were again taken (day 2 post) followed by continued sampling up to day 4 pi. These samples were then analyzed for virus release by plaque assay (as described above in Section 2.2).

### 2.4. Cell Viability Assay

To determine the impact on cell viability of the drug concentrations used in our experiments, Vero76 cells were seeded with a confluence of 80–90% in white 96-well plates. They were then treated with each of the compounds either early or late, as described above in Section 2.3, with the exception that the cells were not infected in this assay. As a positive control for cell death, staurosprorine treatment (STS; 10 μM, Abcam, Cambridge, UK) was used for 48 h. At the indicated time points, cell viability was measured using the ATPlite 1step Luminescence Assay System (Perkin–Elmer, Waltham, MA, USA) according to the manufacturer’s protocol. Briefly, the ATPlite 1step reagent was added to the wells in a 1:3 ratio and the plate was incubated on a shaker for 2 min at 700 rpm. Additional wells did not receive the reagent in order to estimate the background signal from the cells. The white plate was adapted to darkness for 10 min before luminescence was measured using a Glomax Multi multiplate reader (Promega, Madison, WI, USA).

### 2.5. Western Blot

Infected cells were harvested from 12-well plates by scraping them into the culture medium, after which they were centrifuged at 6000× *g* for 5 min at 4 °C and lysed for 40 min on ice in cell extraction buffer (CEB, Invitrogen/Thermo Fisher Scientific, Waltham, MA, USA) supplemented with 1× cOmplete Protease Inhibitor Cocktail (Sigma–Aldrich) and 1 mM phenylmethylsulfonylfluoride (Sigma–Aldrich). After lysis, cell debris was centrifuged down at 18,000× *g* for 10 min at 4 °C and the obtained protein lysates were mixed with 4× SDS gel loading buffer (10% SDS (*w*/*v*), 40% glycerol (*v*/*v*), 20% β-mercaptoethanol, 0.008% bromophenol blue, 250 mM Tris-HCl pH 6.8) and heated twice at 99 °C for 10 min. Samples were separated on 12.5% polyacrylamide gels by electrophoresis (Bio-Rad, Hercules, CA, USA) and transferred to Odyssey nitrocellulose membranes (LI-COR Biosciences, Lincoln, NE, USA) by semi-dry blotting at 15 V for 90 min. Blots were blocked in 7% skim milk prepared in Tris-buffered saline containing 0.01% Tween-20 (TBS-Tween) for 60 min at room temperature. Antibodies were diluted in 1% skim milk in TBS-Tween at the concentrations indicated below in Section 2.6. Primary antibodies were incubated overnight at 4 °C, whereas secondary antibodies were incubated for 60 min at room temperature. TBS-Tween was used to wash membranes thoroughly between all incubations, except for the final washing, which was performed twice in TBS without Tween. Fluorescence signals were detected using the Odyssey Imager (LI-COR) and quantified using the Image Studio Lite Version 5.2 software (LI-COR). For the detection of Bad, chemiluminescence signals were detected using Clarity Western Substrate (Bio-Rad) on X-ray film (Fuji Super RX-N 13 × 18, Fujifilm, Minato, Tokyo Prefecture, Japan). The data shown for each protein, as well as for treated and untreated samples, are from the same gels, but are shown separated for clarity.

### 2.6. Antibodies

Primary antibodies from Cell Signaling Technology (Danvers, MA, USA) were used to detect Caspase 3 (#9662; 1:1000/1:500 [full-length/cleaved]), Bad (#9239; 1:500), phospho-Bad (Ser112) (#5284; 1:500), Erk (#4695; 1:1000), phospho-Erk (#9101; 1:1000), Akt (#9272; 1:1000), phospho-Akt (#4060; 1:200), MK2 (#3042; 1:500), phospho-MK2 (Thr222) (#3316; 1:200), phospho-MK2 (Thr334) (#3007; 1:200), p38 (#9212; 1:1000), phospho-p38 (#4511; 1:2000), JNK (#9252; 1:1000), phospho-JNK (#9251; 1:500), c-Jun (#9165; 1:1000) and phospho-c-Jun (#3270; 1:1000). Primary antibodies against p53 (sc-47698; 1:1000), phospho-p53 (Ser392) (sc-51690; 1:500) and Vinculin (sc-73614; 1:1000) were obtained from Santa Cruz Biotechnology (Dallas, TX, USA). Polyclonal antibodies against TCRV (1:1000) and JUNV (1:1000) NP (produced in guinea pigs [15]) were used to confirm viral infection. Fluorescently labeled secondary antibodies were purchased from LI-COR (IRDye 680RD Donkey anti-Mouse IgG, IRDye 680RD Donkey anti-Guinea Pig IgG, IRDye 800CW Donkey anti-Rabbit IgG) and used at a dilution of 1:15000. A goat anti-rabbit IgG HRP-linked secondary antibody (#7074; 1:5000, Cell Signaling Technology) was used for the detection of Bad.

### 2.7. Statistical Analysis

Quantification data represent the means and standard deviations from multiple independent experiments, as indicated in the figure legends for the individual assays. Statistical analysis was performed using GraphPad Prism version 9.3.1, with differences between groups analyzed using two-way ANOVA using Sidak’s post hoc test (comparison of selected pairings). Significance cut-offs were defined as * *p* ≤ 0.05; ** *p* ≤ 0.01; *** *p* ≤ 0.001; **** *p* ≤ 0.0001.

## 3. Results

**Figure 1:**
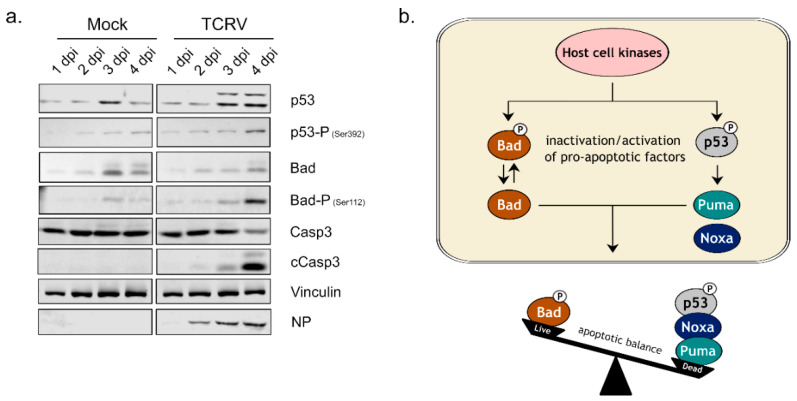
Regulation of pro-apoptotic factors during TCRV infection and its consequences for cell fate: (**a**) Vero76 cells were mock-infected or infected with TCRV (MOI = 0.1) and cell lysates were collected 1–4 days post-infection. Western blotting was performed to detect protein levels for p53, phosho-p53 (Serine 392), Bad, phospho-Bad (Serine 112), the full-length form of caspase 3 (Casp3) and its cleavage products (cCasp3), as indicated. Staining for Vinculin was used as a loading control and detection of NP served as a control for infection; (**b**) schematic model of the host cell factors involved in the regulation of apoptotic balance during TCRV infection. Yet unknown host cell kinases become activated in response to viral infection and subsequently mediate phosphorylation of Bad and p53. This leads to the inactivation of the pro-apoptotic factor Bad and the activation of p53, which then mediates transcription of the pro-apoptotic factors Puma and Noxa. These factors act as competing forces in the regulation of apoptotic balance within the infected cell, which late in infection favors triggering downstream apoptotic processes that ultimately lead to caspase activation and result in cell death.

We have previously shown that TCRV infection leads to the activation of the intrinsic apoptotic pathway and, in particular, phosphorylation of pro-apoptotic factors known to regulate the apoptotic balance in stressed cells [17]. Indeed, following infection of Vero76 cells with TCRV, we were able to confirm the phosphorylation of the tumor suppressor protein p53 (at Serine 392) and the BH3-only factor Bad (at Serine 112), both of which become prominent at day 4 pi, which is also consistent with the kinetics of caspase 3 cleavage in response to infection (Figure 1a). We have also previously shown that this activation of p53 leads to its stabilization and nuclear translocation, with subsequent transcription of the pro-apoptotic BH3-only factors Puma and Noxa [19,34,35] (Figure 1b). In contrast, the phosphorylation of Bad results in the inactivation of its pro-apoptotic function [21] and shifts the apoptotic balance to delay cell death (Figure 1b). This role of p53 and Bad phosphorylation in regulating this process clearly indicates the involvement of upstream host cell kinase activation in triggering apoptotic signaling (Figure 1b).

### 3.1. Activation of Kinase Signaling Pathways by TCRV Infection

**Figure 2:**
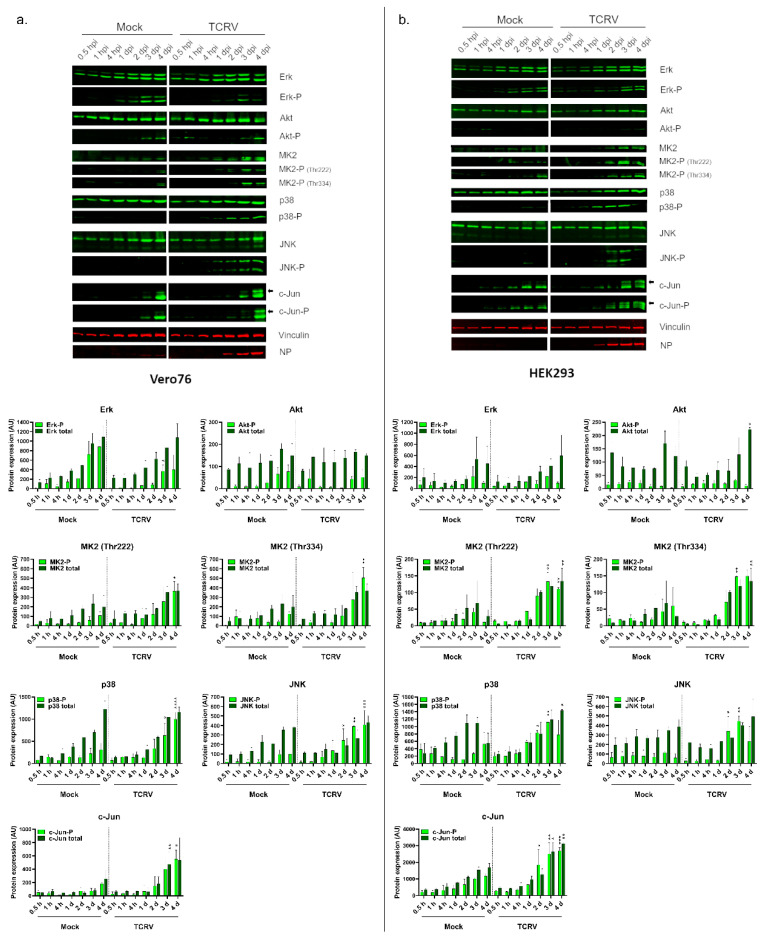
TCRV affects the expression and activation of diverse kinases. Kinase expression and activation in Tacaribe virus-infected (**a**) Vero76 cells and (**b**) HEK293 cells. Cells were infected at an MOI of 0.1 and cell lysates were harvested at the indicated time points post infection for analysis by Western blot with specific antibodies for Erk, phosho-Erk, Akt, phosho-Akt, MK2, phosho-MK2 (Threonine 222), phosho-MK2 (Threonine 334), p38, phosho-p38, JNK, phosho-JNK, c-Jun and phosho-c-Jun, as indicated. Black arrows indicate the activated form of c-Jun which shows a shift in its apparent molecular weight. Mock-infected cells served as a negative control and staining for vinculin was used as a loading control. Detection of the viral nucleoprotein (NP) was used to demonstrate infection. For quantification (lower panels) pixel intensities for total and phosphorylated protein bands were measured using the LI-COR system. The means and standard deviations of at least two independent experiments are shown. Statistical significance was determined using two-way ANOVA (* *p* ≤ 0.05, ** *p* ≤ 0.01, *** *p* ≤ 0.001, **** *p* ≤ 0.0001).

To shed further light on early steps in the regulation of apoptosis induction during TCRV infection, we therefore examined the activation of the major kinase signaling pathways associated with the regulation of apoptosis (i.e., Erk, Akt, p38, JNK), as well as some of their downstream targets (i.e., MK2 and c-Jun) [22,36]. Based on our previous work, the activation of apoptosis regulating proteins (e.g., p53 and Bad) by phosphorylation appears to be common among different cell types (including primary human monocytes [17]). Nonetheless, we performed these infections both in Vero76 cells (Figure 2a), where most of our work on the regulation of apoptosis has been carried out, as well as a human cell line, HEK293 (Figure 2b). As expected, these cell lines showed very similar patterns of activation for the SAPKs (i.e., p38, JNK), as well as activation of their downstream targets MK2 and c-Jun. Specifically, TCRV infection led to a significantly increased phosphorylation of both p38 and JNK in both cell lines starting from day 2 pi and increasing over the time (Figure 2a,b). However, for both factors these signals disappeared again in HEK293 cells at day 4 pi (Figure 2b), presumably due to the accumulation of dead cells as a result of apoptosis. We could also show phosphorylation of the p38-regulated downstream kinase MK2 on the residues Threonine 222 and 334 (at day 3 and 4 pi) as well as an increase in total MK2 expression (Figure 2a,b). Similarly, for the JNK-regulated transcription factor c-Jun, both phosphorylation and total protein levels also increased over the duration of the infection (Figure 2a,b).

Interestingly, however, there were marked differences in the regulation of pro-survival kinases between Vero76 and HEK293 cells (c.f. Figure 2a,b). For instance, while TCRV-infection of both cell lines resulted in accumulation of phosphorylated Erk on day 3 and 4 pi, in Vero76 cells these levels were still reduced compared to mock-infected cells (Figure 2a). In contrast, HEK293 cells showed comparable levels of both total and phospho-Erk between TCRV- and mock-infected samples (Figure 2b). These cell lines also showed distinct patterns of regulation for Akt. Specifically, while infection in Vero76 cells resulted in an early activation of Akt (i.e., at 1 h pi), this could not be observed in HEK293 cells. Increased levels for phospho-Akt seen in Vero cells at late time points are similar between mock- and TCRV infected samples.

Taken together, these results indicate that TCRV induces activation of two SAPKs (i.e., p38 and JNK) that are known to regulate pro-apoptotic signaling, as well as activation of their downstream targets such as MK2 and c-Jun. Moreover, these kinases showed strong activation late in the infection, which is consistent with the observed induction of apoptotic signaling events in these cells at these time points (c.f. Figure 1a). In contrast, TCRV infection does not induce activation of the pro-survival kinases Erk and Akt late in the infection (i.e., at time points when apoptotic signaling events are occurring). Nevertheless, an early activation of Akt was also observed in Vero76-infected cells, supporting a possible involvement of Akt regulation in earlier steps during the viral life cycle, even if it is unlikely to play a role in the regulation of apoptosis.

### 3.2. Inhibition of Kinase Activation Significantly Reduces Viral Growth

Given that we detected clear evidence for the activation of several different kinases, as well as their downstream targets during TCRV infection, we further sought to clarify whether this regulation is of relevance for productive viral infection. To do so, we used specific chemical inhibitors and activators that target p38, JNK, MK2, c-Jun, Akt or Erk, which we applied during distinct phases of the infection (i.e., early or late) to assess their impact on viral growth.

**Figure 3:**
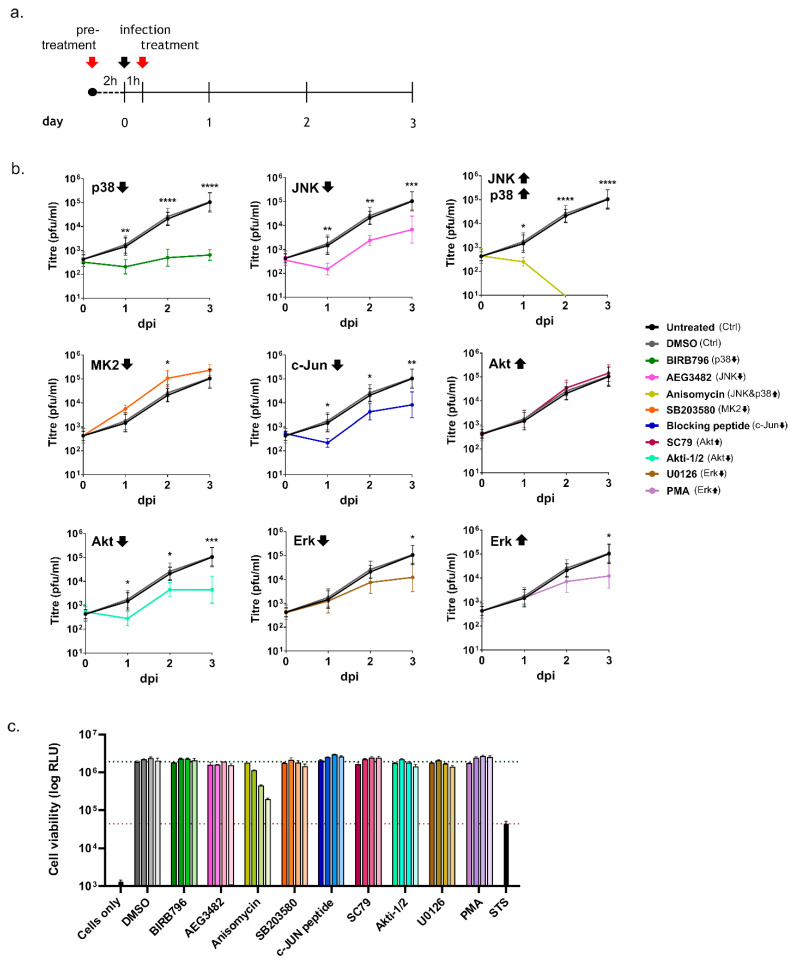
Influence of kinase inhibition or activation early in the infection on TCRV growth: (**a**) schematic timeline of the chemical treatment and infection procedure. Red arrows indicate the addition of drug dilutions and the black arrow indicates the start of the infection. Supernatants were harvested from day 0 to day 3 post infection; (**b**) virus growth. Vero76 cells were infected at MOI = 0.1 and treated with the indicated chemical compounds according to the scheme shown in (**a**). Samples were collected on days 0–3 post infection for analysis by plaque assay. Specific inhibitors for p38 (BIRB796; green), MK2 (SB203580; orange), Akt (Akti-1/2; turquoise), JNK (AEG3482; pink), c-Jun (c-Jun blocking peptide; blue) and Erk (U0126; brown), as well as specific activators for p38 and JNK (Anisomycin; dark yellow), Akt (SC79; magenta) and Erk (PMA; purple) were used. Infected untreated (black) and DMSO-treated (gray) cells served as controls. Statistical analysis was performed using two-way ANOVA (* *p* ≤ 0.05, ** *p* ≤ 0.01, *** *p* ≤ 0.001, **** *p* ≤ 0.0001). The data shown are the mean values and standard deviations from three independent experiments; and (**c**) cell viability assays. Cells treated with the indicated compounds were assessed for cell viability using the ATPlite 1step Luminescence Assay at the time points indicated in (**a**). The color code corresponds to that used for the different drugs in (**b**) with progressively lighter shades of the corresponding colors indicating the different time points, at which cell viability was measured (day 0 to day 3 post treatment). Black bars represent control cells that were either not incubated with the ATPlite reagent (cells only) or treated with 10 μM STS to induce cell death as a positive control. The data shown represent means and standard deviations from two independent experiments.

In the first experiment, we performed a pre-treatment prior to infection with TCRV, followed by treatment with fresh drug dilutions following the removal of the inoculum (Figure 3a). While DMSO-treated cells supported the growth of TCRV at levels similar to untreated control cells, treatment with several of the chemicals tested led to drastic changes in viral titres (Figure 3b). In particular, early inhibition of p38, JNK, c-Jun, and Akt resulted in markedly reduced viral replication compared to control cells, supporting a biological relevance for the observed activation of these kinases during infection (Figure 3b). In contrast, blocking MK2 activity resulted in the opposite effect and slightly increased viral multiplication at day 2 pi (Figure 3b). Surprisingly, the early inhibition or activation of Erk led to only a very modest drop in viral titres that was only detectable on day 3 pi, however the effect was limited compared to inhibition of the other kinases (Figure 3b). Interestingly, the other activators used in this study showed either no effect on infection kinetics, i.e., for Akt activation, or rather an inhibitory impact on viral replication, as shown for Erk and for JNK/p38 activation (Figure 3b). Importantly, however, the dramatic loss of viral replication observed during treatment with the JNK/p38 activator anisomycin can most likely be explained by its pronounced effects on cell viability (Figure 3c), consistent with the fact that JNK and p38 are mostly responsible for triggering pro-apoptotic signaling in stressed cells as well as the fact that anisomycin is also known to be a potent inhibitor of protein synthesis. In contrast, treatment with the other drugs seemed to have little or no negative impact on cell viability over the timeframe and at the dilutions used in our study (Figure 3c), supporting the notion that our observations for these kinases are due to the blocked/activated kinase activities rather than cytotoxic effects induced by the drug treatments.

**Figure 4:**
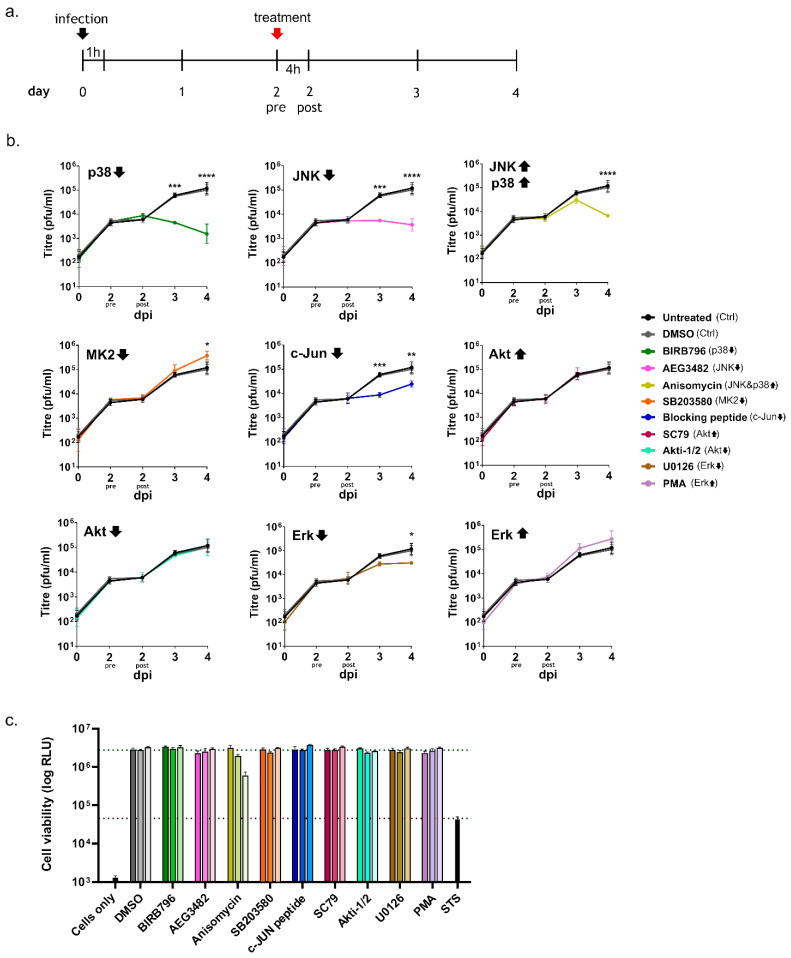
Influence of kinase inhibition or activation late in the infection on TCRV growth: (**a**) schematic timeline of the chemical treatment and infection procedure. The black arrow indicates the onset of the infection and the red arrow indicates the addition of drug dilutions on day 2 post infection. Supernatants were harvested from day 0 to day 4 post infection. Samples were taken twice on day 2 post infection—before adding the drugs (2 pre) and 4 h after the treatment (2 post); (**b**) virus growth. Vero76 cells were infected at MOI = 0.1 and treated with the indicated chemical compounds according to the scheme shown in (**a**). Samples were collected on days 0, 2, 3 and 4 post infection for analysis by plaque assay. Specific inhibitors for p38 (BIRB796; green), MK2 (SB203580; orange), Akt (Akti-1/2; turquoise), JNK (AEG3482; pink), c-Jun (c-Jun blocking peptide; blue) and Erk (U0126; brown), as well as specific activators for p38 and JNK (Anisomycin; dark yellow), Akt (SC79; magenta) and Erk (PMA; purple) were used. Infected untreated (black) and DMSO-treated (gray) cells served as controls. Statistical analysis was performed using two-way ANOVA (* *p* ≤ 0.05, ** *p* ≤ 0.01, *** *p* ≤ 0.001, **** *p* ≤ 0.0001). The data shown are the mean values and standard deviations from three independent experiments; (**c**) cell viability assays. Cells treated with the indicated compounds were assessed for cell viability using the ATPlite 1step Luminescence Assay at the time points indicated in (**a**). The color code corresponds to that used for the different drugs in (**b**) with progressively lighter shades of the corresponding colors indicating the different time points, at which cell viability was measured (4 h post treatment (corresponding to the post-treatment day 2 samples) to 2 days post treatment (corresponding to day 4 samples)). Black bars represent control cells that were either not incubated with the ATPlite reagent (cells only) or treated with 10 μM STS to induce cell death as a positive control. The data shown represent means and standard deviations from two independent experiments.

In a further experiment, we infected Vero76 cells and added kinase inhibitors or activators only starting on day 2 pi, in order to assess their impact late during infection, i.e., once a productive infection had been established (Figure 4a). Interestingly, and consistent with the effects seen following early treatment, adding inhibitors of p38, JNK or c-Jun on day 2 pi also significantly impaired viral replication on subsequent days, i.e., 3 dpi and 4 dpi (Figure 4b). In contrast, unlike an early inhibition of Akt, which led to strikingly reduced replication, adding the Akt inhibitor later during infection did not influence viral growth (Figure 4b). This further supports the idea that Akt might have an important role in regulating early events during infection and is consistent with our observation that, unlike these other kinases, it is only transiently upregulated early post infection (c.f. Figure 2a). We also observed that the inhibition of Erk activity had much less of an effect when performed late in the infection, and only resulted in a slight decrease in virus titre on day 4 post infection (Figure 4b). However, similar to what we observed for early inhibition of MK2 inhibition, late inhibition of this kinase also resulted in slightly increased viral replication (Figure 4b). As for the experiments examining early addition, the late addition of activators for Akt and Erk did not significantly influence TCRV growth (Figure 4b). At late time points, the activation of p38/JNK by treatment with anisomycin led to a drastic drop in viral titres (Figure 4b); however, this treatment regime was also accompanied by a dramatic loss of cell viability (Figure 4c). Late treatment with other chemicals did not impair cell viability at the concentrations used in our experiments (Figure 4c).

In summary, these data show that TCRV replication is clearly suppressed upon specific inhibition of p38, JNK or the downstream transcription factor c-Jun, and that this is independent of when in the infection the drugs were applied, implying a more general pro-viral role of these factors in supporting viral infection. However, significant differences were observed for the inhibition of Akt, which only impacted viral growth early during infection, supporting a possible role in entry-related processes. In contrast, a slight increase in viral titres could be observed following MK2 inhibition, indicating that it has a modest antiviral role during TCRV infection. Taken together, these data clearly suggest that while a number of different kinases are important for the success of the viral infection, they are involved in regulating distinct stages of the viral life cycle.

## 4. Discussion

Highly pathogenic arenaviruses pose a significant threat for public health in many areas of the world, but there are only limited antiviral therapies available [37]. Furthermore, while the live attenuated JUNV vaccine Candid#1 produces an effective and long-lasting immunity, no similar vaccines have so far been approved for other related HF-causing arenaviruses [38,39,40]. The identification of novel arenaviruses in recent years, including those associated with human disease (e.g., Lujo virus and Chapare virus [41,42]) emphasizes the need for broad-spectrum approaches for the management of arenaviral infections. A promising approach to the development of such antiviral strategies is targeting conserved host cell factors that are important either for supporting viral infection or play a role in regulating the antiviral response to viral infection (reviewed in [43]). In this respect, host cell kinases represent particularly interesting therapeutic targets, as many specific inhibitory compounds are already available for the management of non-viral disease (e.g., cancer) and have thus been shown to meet the safety requirements needed for clinical utility [44]. Promising kinase inhibitors have also been described for the treatment of SARS-CoV-2 infection, where they act to suppress hyperinflammatory responses, further highlighting the therapeutic potential to combat other severe viral diseases using these kinds of approaches [45].

**Figure 5:**
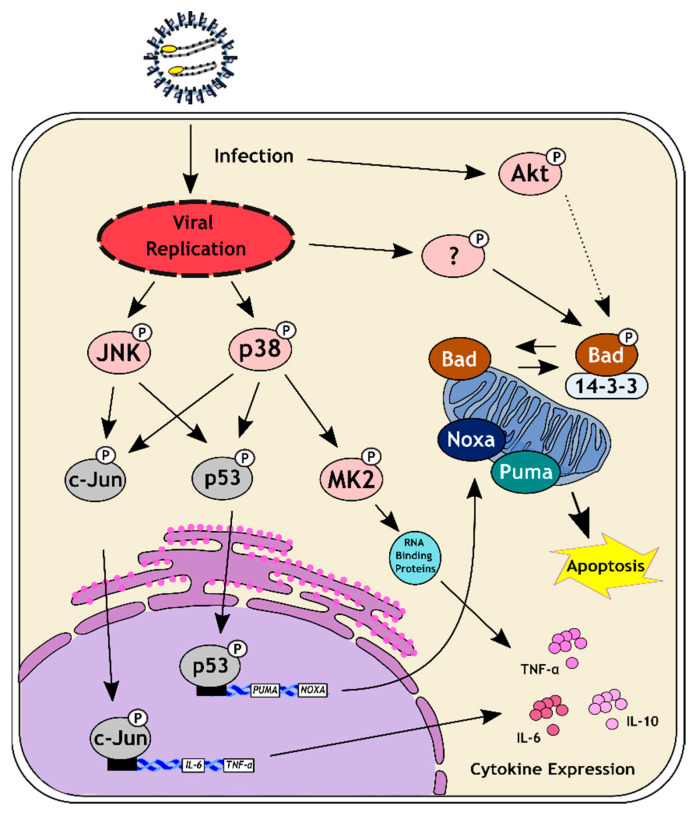
Model for the role of kinase signaling in the regulation of apoptosis induction and the cytokine response to TCRV infection. TCRV triggers transient phosphorylation/activation of the pro-survival kinase Akt very early after infection. While in other contexts this kinase is known to trigger phosphorylation of the pro-apoptotic factor Bad (leading to its inactivation through sequestration by the 14-3-3 protein), the timing of its activation does not coincide with other apoptosis-related signaling events. This suggests that the phosphorylation of Bad observed during TCRV infection [17] must be mediated by another yet unknown kinase. Once productive infection has been established, TCRV infection also results in phosphorylation/activation of the stress-activated protein kinases p38 and JNK. This coincides with other pro-apoptotic signaling events (i.e., p53 phosphorylation, expression of Puma and Noxa) [17], suggesting a potential role for these pro-apoptotic kinases in activating TCRV-mediated apoptosis. Downstream targets of p38 and JNK, which include the kinase MK2 and the transcription factor c-Jun, are also activated late during infection. These factors are also known to regulate apoptotic processes. However, they are associated with cytokine expression as well, either directly by acting as a transcription factor (i.e., c-Jun), or indirectly by activating RNA-binding proteins that stabilize cytokine mRNA transcripts, and particularly those shown to be upregulated during TCRV infection of primary target cells (i.e., TNF-α, IL-6, IL-10) [11].

Excitingly, we have observed that the regulation of apoptosis induction during arenavirus infection is controlled by the phosphorylation of specific pro-apoptotic factors (i.e., Bad and p53 phosphorylation (Figure 1) [17], clearly suggesting a role for kinase activation in this process, and also suggesting other possible links to host cell responses important for the outcome of viral infection and pathogenesis (Figure 5). As such, we investigated the activation status of different upstream signaling pathways that have been previously reported to be linked to the phosphorylation events responsible for TCRV-induced apoptosis in order to better understand their possible involvement in these processes.

We were able to detect strong phosphorylation for both p38 and JNK (Figure 2) late in TCRV infection. This is consistent with the timeframe during which accumulation and phosphorylation of p53 occur, as well as classical hallmarks of apoptosis, such as visible morphological changes (i.e., cell death), loss of membrane asymmetry and caspase activation [15,17]. Importantly, both of these kinases are known to phosphorylate p53, thereby inducing its stabilization and activation [19,20,28]. Interestingly, although the highly pathogenic JUNV does not induce caspase activation or apoptotic cell death, due to its ability to use its nucleoprotein (NP) as a decoy substrate for caspase cleavage [18], we have previously shown that similar upstream pro-apoptotic signaling events (i.e., stabilization and phosphorylation of p53) are also occurring [17]. Consistent with this, we also saw phosphorylation of both p38 and JNK during JUNV infection (Supplementary Figure S1). Surprisingly, recent data have indicated that the JUNV vaccine strain Candid#1 also induces apoptosis, and that this is related to improper processing of the glycoprotein, due to changes in its glycosylation, leading to retention in the ER [46]. This appears to result in activation of the unfolded protein response (UPR) [47], a cellular mechanism that can also trigger apoptosis though activation of JNK under conditions where ER stress remains unresolved [48]. Thus, while the activation of JNK in response to Candid#1 infection remains to be formally demonstrated, these commonalities among NW arenaviruses suggest a conserved role of SAPK activation in initiating apoptosis during arenavirus infection.

Indeed, an important role for p38 and JNK during TCRV infection is supported by our data, which showed a strong antiviral effect of their inhibitors regardless of whether activity of these kinases was blocked early (i.e., prior to entry) or late (i.e., after productive infection has been established) during the infection. This observation is consistent with the reported effects of p38 inhibition in HEK293 and Vero cells during JUNV XJ13 infection, where viral growth was successfully inhibited when the drugs were applied in an early stage of the infection [32]. In contrast, the regulation and participation of JNK signaling in arenavirus infection has not been previously investigated. We also observed significant loss of viral replication in cells treated with the JNK and p38 activator anisomycin; however, treatment was accompanied by high levels of cell death. This is potentially consistent with both the role of JNK and p38 in inducing apoptotic cell death and the known effects of anisomycin on important cellular processes, such as the inhibition of eukaryotic protein biosynthesis [49]. Unfortunately, this makes it impossible to assess the specific contribution of p38/JNK activation to anisomycin’s effect on viral infection. Nonetheless, the observed activation of both p38 and JNK, taken together with the inhibitor data strongly suggest a pro-viral role for these kinases during arenavirus infection. Initially, such a finding is somewhat surprising, given that the induction of apoptosis itself does not have any direct pro- or antiviral effects on TCRV infection [15,17], and suggests that p38 and JNK inhibition are also contributing to other processes of relevance to virus growth.

In this regard, it is worth noting that p38 and JNK are not only involved in mediating apoptotic signaling, but also have important immune functions due to their role in controlling downstream factors that either directly or indirectly contribute to cytokine production. In particular, the p38-regulated kinase MK2 can directly affect the stability of cytokine mRNA transcripts through the regulation of RNA-binding proteins that interact with AU-rich elements in the mRNA 3′-untranslated region [50], while JNK activation contributes to the activation of AP-1 family transcription factors, e.g., c-Jun, that also contribute to cytokine mRNA expression [51,52,53] (Figure 5). Therefore, since we were able to detect activation of both JNK and p38, we also investigated the downstream activation of MK2 and c-Jun during TCRV infection—and, indeed, we saw activation of both of these factors late in the infection, consistent with the kinetics of JNK and p38 activation (Figure 2), and also the regulation of apoptotic regulatory factors (Figure 1) [17]. Intriguingly, regulation by MK2 and c-Jun is known to specifically effect those cytokines that have been previously shown to be upregulated during TCRV infection of primary monocytes and macrophages (i.e., TNF-α, IL-6 and IL-10) [11]. Interestingly, however, consistent with our data for the activation of JNK and p38, we also saw MK2 and c-Jun activation late during infection with the virulent JUNV strain (Supplementary Figure S1). While virulent JUNV does not produce these cytokines during infection in monocytes and macrophages [11], elevated levels of these same factors are hallmarks of the cytokine storm that accompanies severe cases of AHF in patients [9]. Consequently, these data suggest that these factors may indeed play an important role in regulating cytokine induction in response to NW arenavirus infection, but they also speak for a more complicated and also context-dependent relationship that is likely also influenced by other signaling molecules/pathways as well. Interestingly, we also saw very different effects of MK2 and c-Jun inhibition on TCRV infection (Figure 3 and Figure 4). Specifically, while c-Jun inhibition resulted in a significant drop in viral replication (comparable to that seen following JNK inhibition), blocking MK2 activity led to slightly increased viral titres. This is in contrast to the inhibition of its direct upstream factor p38, and suggests a distinct antiviral role for MK2. Consequently, it will be important to expand these studies to primary human monocytes and/or macrophages (or other immune cells), where differences in the regulation of c-Jun and MK2 may play a more relevant role in the regulation of cytokine expression profiles during viral infection.

Interestingly, unlike for the SAPKs, we did not observe activation of the pro-survival kinase Erk compared to mock-infected cells in TCRV-infected Vero76 or HEK293 cells at any of the time points examined (Figure 2). Specifically, while levels of phospho-Erk did increase late during infection, signals in mock-infected cells increased to a similar (or in the case of Vero cells even greater) extent, suggesting that this is not a specific virus-induced activation. Similarly, following infection with the highly pathogenic JUNV strain Romero we also saw no evidence of Erk activation (Supplementary Figure S1). Nonetheless, inhibition or activation of Erk prior to infection showed a modest impact on replication, indicating that this factor does play a role in supporting an early phase of viral infection. In contrast, late treatment had little if any impact on viral infection. On the one hand, this supports previous work showing that chemical inhibition/activation of Erk activity during infection with JUNV XJ13, TCRV and PICV affects virus growth [24]. However, in contrast to these published data, we did not see direct evidence of a transient Erk activation in response to the infection itself. This was despite using the same chemicals, a related cell line (i.e., another Vero cell line) and including similar time points. These studies also used similar concentrations of these drugs, which, as we show here, do not significantly impact cell viability (Figure 3 and Figure 4). One further possible explanation for this difference that we considered was that these previous experiments were performed under conditions of serum starvation, which represents an additional stress being imposed on these cells that could affect pro-survival kinase signaling [54,55]. Therefore, to be able to more directly compare our results with the previously published data, we also performed infections with TCRV under these altered experimental conditions; however, increased Erk phosphorylation in response to infection still could not be detected (Supplementary Figure S2). Thus, it remains unclear at the moment under what conditions Erk activation in response to infection actually occurs, and thus what role it plays, although as noted above, our data do support a biological role of Erk activity in supporting viral infection. Nonetheless, in light of our specific research question, the lack of a specific infection-induced Erk activation in our experiments suggests that, at least under the experimental conditions used in our study, another kinase is likely responsible for the observed Bad phosphorylation and its role in the regulation of TCRV-induced apoptosis.

Another candidate that we examined in connection with such a function was Akt, which is also known to regulate the pro-apoptotic activity of Bad by phosphorylation [21]. Indeed, we did observe weak infection-induced Akt phosphorylation in response to both TCRV (Figure 2a) and JUNV infection (Supplementary Figure S1), but this was only the case at early times post infection (i.e., 30–60 min post infection). This is in contrast to the regulation of pro-apoptotic factors, which occurs rather late in TCRV infection, consistent with the accumulation of cleaved caspases at these time points (Figure 1a) and also the late onset of apoptotic cell death [15,17]. Indeed, similar to Erk phosphorylation, while we do see increased levels for phospho-Akt occurring on days 3 and 4, this is the case in both TCRV- and mock-infected cells. Again, this suggests that TCRV does not induce a specific late activation of this kinase, and highlights the importance of appropriate time-matching mock controls in studies of kinase activation. In accordance with these observations, only early chemical inhibition of Akt significantly impaired TCRV growth, indicating that this kinase rather plays a crucial role for early events in the virus lifecycle. This supports previously published data for JUNV XJ13 infection, in which Akt activation was detected 15–30 min post infection and where early inhibition of Akt also led to impaired viral replication [29]. From a mechanistic standpoint, that study further suggested that virus internalization was responsible for Akt activation, comparable to what has been shown for Akt activation upon transferrin treatment, a ligand that binds the human transferrin receptor, which is used by JUNV for viral entry [56,57]. A possible connection between Akt activation and transferrin receptor recycling, which is necessary for optimal virus binding has been hypothesized for JUNV XJ13, as well [29]. This makes it somewhat surprising that we also observe modest early Akt activation during TCRV infection (and that it is still important for viral growth), since TCRV that does not use the human transferrin receptor for viral entry. As such, our data appear to suggest a broader role for Akt in arenavirus entry that needs to be further examined.

Taken together, we were able to identify several host cell signaling factors that are clearly activated during infection with TCRV. Importantly, several of these factors, i.e., p38 and JNK, are known to be associated with p53 phosphorylation, and which we thus propose may fulfil a similar role during the p53-mediated induction of apoptosis in response to TCRV infection (Figure 5). Intriguingly, downstream activation of the p38 and JNK-regulated factors MK2 and c-Jun suggests a further possible role for the activation of these pathways in the regulation of cytokines previously described to be differentially regulated during TCRV and JUNV infection [11] (Figure 5) that needs to be further explored. In contrast, our data do not support a role of the pro-survival kinases Erk or Akt in the regulation of apoptosis (e.g., through Bad phosphorylation) given that we either did not see activation of these factors over levels seen in mock samples (i.e., Erk) or their activation was restricted to early time points inconsistent with the kinetics of TCRV-induced apoptosis (i.e., Akt). Thus, further work is now needed, not only to confirm the direct role of p38 and JNK in p53 activation, but also to examine the activation of other signaling pathways known to regulate Bad phosphorylation, that might then also be involved in regulating this aspect of TCRV-induced apoptosis. Candidates include cAMP-dependent protein kinase (PKA) or p90 ribosomal S6 kinase (RSK) [58,59]. At the same time, another important area of future work will be to more precisely define the role that these kinases play in supporting viral infection. Nonetheless, together with other previously published data [23,24,25,29,30,31,32,33,60], our findings already clearly support a critical role of several kinases, including SAPKs, in arenavirus infection, making these signaling pathways promising potential targets for the development of broad-spectrum antiviral therapies. Indeed, this may be particularly feasible from a practical standpoint, since many kinase inhibitors are already clinically approved and can be used in the management of cancer (reviewed in [44]) as well as for some viral infections (e.g., in HIV infection), while others are under clinical/preclinical investigation for treatment of COVID-19 (reviewed in [43,45]).

## Figures and Tables

**Table 1:** Host cell kinase inhibitors and activators used for growth kinetics.

Target	Chemical Name	Supplier	Final Conc.
Akt ↓	Akti-1/2	Tocris # 5773	20 µM
Akt ↑	SC79	Tocris # 4635	30 µM
Erk↓	U0126	Tocris # 1144	30 µM
Erk ↑	phorbol 12-myristate 13-acetate (PMA)	Sigma # P8139	162 nM
JNK ↓	AEG3482	Tocris # 2651	40 µM
p38 ↓	BIRB796	Tocris # 5989	30 µM
JNK/p38 ↑	Anisomycin	Tocris # 1290	1 µM
MK2 ↓	SB203580	Tocris # 1202	30 µM
c-Jun ↓	c-JUN blocking peptide	Tocris # 1989	50 µM

## Data Availability

All data are included in this published article (and its Supplementary Materials).

## Supplementary Materials

The following supporting information can be downloaded at: https://www.mdpi.com/article/10.3390/v14092018/s1, Figure S1: Junín virus selectively affects the expression and activation of diverse kinases. Figure S2: Serum starvation has no impact on Erk activation during Tacaribe virus infection.
