# Supplementary material for: Regulation of Stress-Activated Kinases in Response to Tacaribe Virus Infection and Its Implications for Viral Replication
Source: Viruses. 2022 Sep 12;14(9):2018. doi: 10.3390/v14092018 (PMC9505436; doi:10.3390/v14092018)
Supplement: Supplementary file 1 [file viruses-14-02018-s001.zip › Suppl Figure 1.pdf]

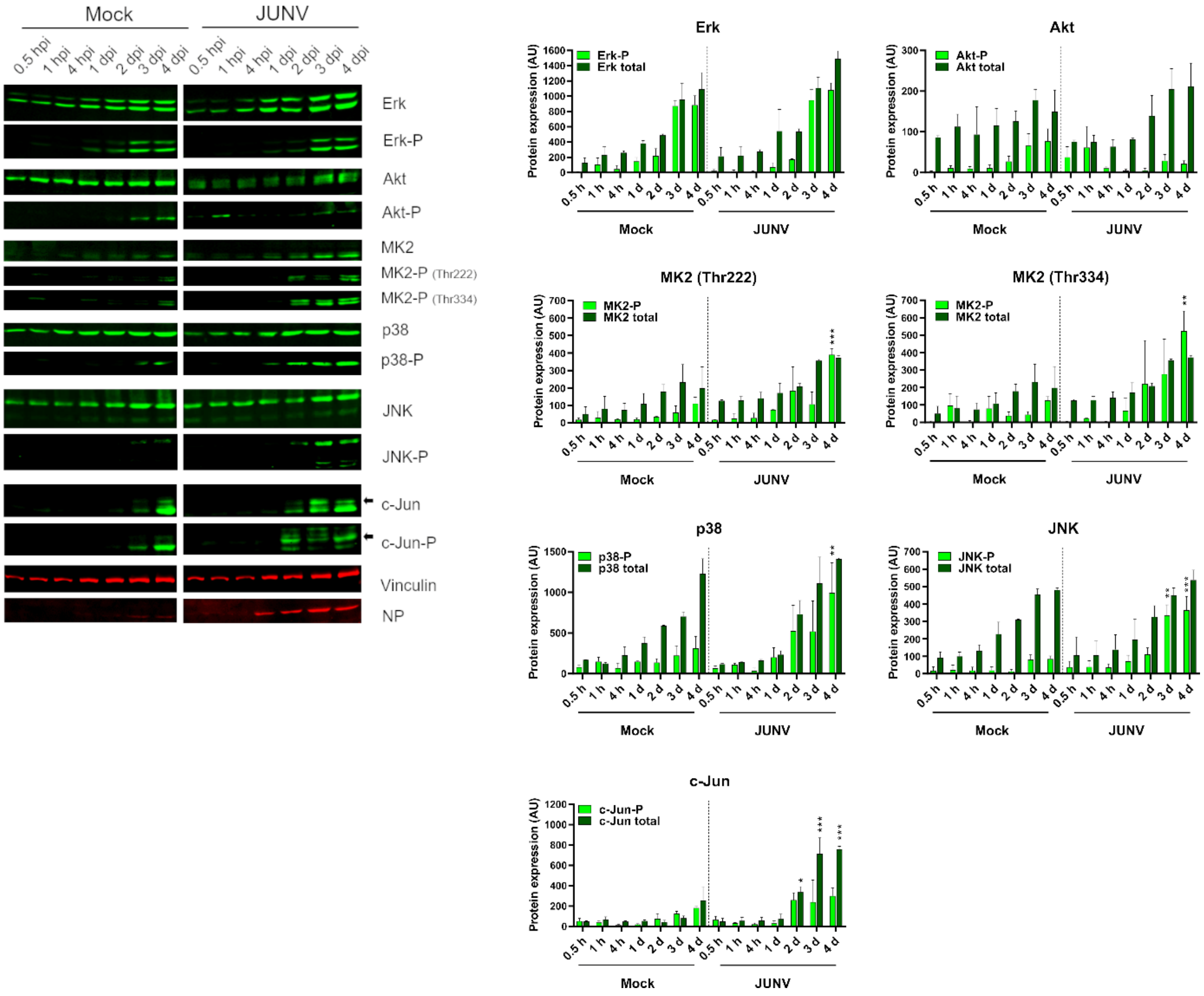

Figure S1. Junín virus selectively affects the expression and activation of diverse kinases. Vero76 cells were infected at an MOI of 0.1 with the virulent Romero strain of JUNV and cell lysates were harvested at the indicated time points post infection for analysis by Western blot with specific antibodies for Erk, phosho-Erk, Akt, phosho-Akt, MK2, phosho-MK2 (Threonine 222), phosho-MK2 (Threonine 334), p38, phosho-p38, JNK, phosho-JNK, c-Jun and phosho-c-Jun, as indicated. Black arrows indicate the activated form of c-Jun which shows a shift in its apparent molecular weight. Mock-infected cells served as a negative control and staining for vinculin was used as a loading control. Detection of the viral nucleoprotein (NP) was used to demonstrate infection. For quantification pixel intensities for total and phosphorylated protein bands were measured using the LI-COR system. The means and standard deviations of two independent experiments are shown. Statistical significance was determined using two-way ANOVA (*p≤0.05, **p≤0.01, ***p≤0.001).
