# Supplementary material for: Regulation of Stress-Activated Kinases in Response to Tacaribe Virus Infection and Its Implications for Viral Replication
Source: Viruses. 2022 Sep 12;14(9):2018. doi: 10.3390/v14092018 (PMC9505436; doi:10.3390/v14092018)
Supplement: Supplementary file 1 [file viruses-14-02018-s001.zip › Suppl Figure 2.pdf]

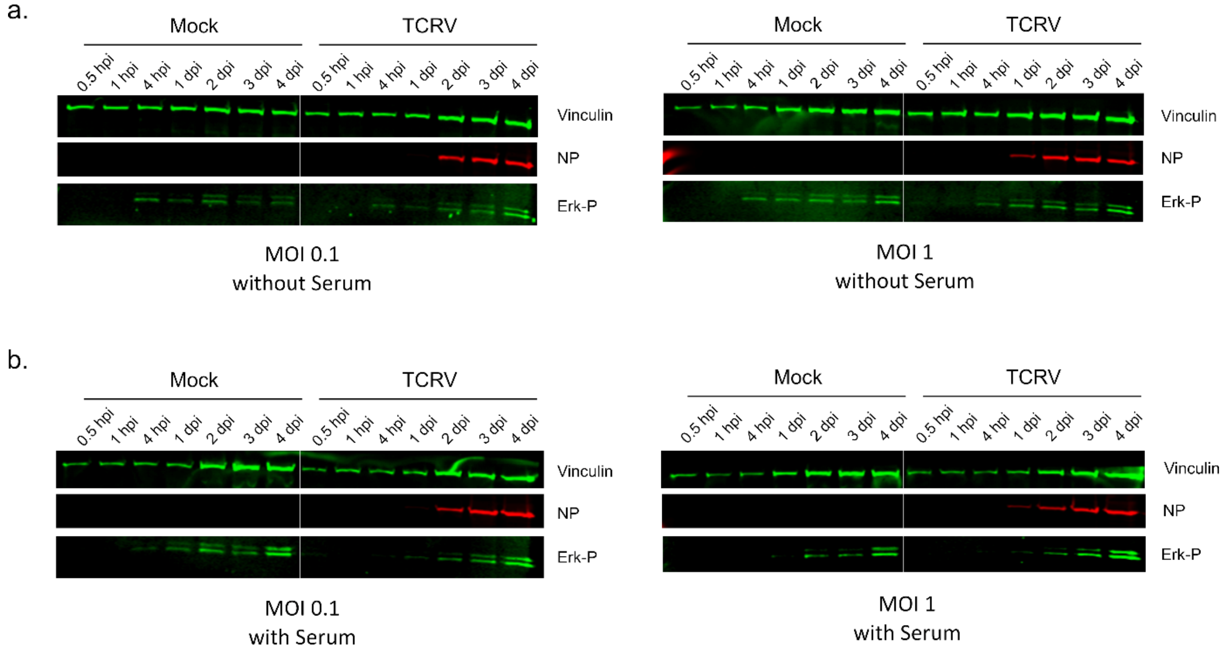

Figure S2. Serum starvation has no impact on Erk activation during Tacaribe virus infection. To compare our results on the phosphorylation status of Erk to that in other recently published work [24, 25], infection was first performed as described in these previous studies. Here (**a**) Vero76 cells were pre-incubated with DMEM containing 0% FCS (without serum) for 1 h prior to infection with an MOI of 0.1 or 1. After removing the inoculum cells were maintained in DMEM without serum. Alternatively, the infection procedure from our own study was used. Here, (**b**) Vero76 cells were TCRV-infected with either an MOI of 0.1 or 1, the inoculum was removed and cells were maintained in DMEM containing 2% FCS (with serum). Cell lysates from both experiments were harvested at the indicated time points post infection for analysis by Western blot with specific antibodies for phosho-Erk, vinculin (loading control) and the viral nucleoprotein NP (control for infection). Mock-infected cells served as a negative control.
